# Editorial Note: Gene/QTL discovery for Anthracnose in common bean (*Phaseolus vulgaris* L.) from North-western Himalayas

**DOI:** 10.1371/journal.pone.0343873

**Published:** 2026-02-27

**Authors:** The PLOS One Editors

The *PLOS One* Editors issue this Editorial Note because this article [1] was identified as one of a series of submissions for which we have concerns about aspects of the peer review process. Readers are advised to interpret the article [1] with caution.

The Editors have no evidence of any author involvement in the peer review concerns.

Additionally, contrary to the declaration in the Data Availability statement, the original raw data files supporting the article’s results were not provided with the article. The authors made these available on editorial request, and the Data Availability statement is therefore updated to: All relevant data are available from https://doi.org/10.6084/m9.figshare.31146682.
